# Charge‐Trap Memory with Engineered Temporal Dynamics for Physically Integrated Reservoir Computing

**DOI:** 10.1002/smsc.202500356

**Published:** 2025-09-29

**Authors:** Mengfan Wu, Ziqi Chen, Niannian Yu, Leyao Li, Xinhao Zhang, Xinyi Wan, Yi Zheng, Shuaishuai Xu, Yang Liu, Jiawei Peng, Yao Wang, Junhui Yuan, Jiafu Wang, Xuewen Wang

**Affiliations:** ^1^ School of Physics and Mechanics Wuhan University of Technology Wuhan 430070 China; ^2^ School of Artificial Intelligence Jianghan University Wuhan 430056 China; ^3^ Center of Femtosecond Laser Manufacturing for Advanced Materials and Devices State Key Laboratory of Advanced Technology for Materials Synthesis and Processing Wuhan University of Technology Wuhan 430070 China; ^4^ International school of Materials Science and Engineering (school of Materials and Microelectronics) Wuhan University of Technology Wuhan 430070 China

**Keywords:** charge‐trap memory, defect engineering, palladium diselenide, reservoir computing, ultrafast photoexcitation

## Abstract

2D material (2DM)‐based reservoir computing (RC) systems combine the advantages of low‐power hardware implementation with lightweight neural network architectures capable of processing complex temporal patterns through minimal training overhead, positioning them as ideal platforms for edge artificial intelligence (AI) applications. Here, a homogeneous RC system via defect engineering in PdSe_2_ charge‐trap memory (CTM) by ultrafast photoexcitation is demonstrated, which directly generates PdSe_2‐x_O_x_ nanodefects, converting volatile states (≈0% retention) into nonvolatile states (≈80% retention) by introducing electron‐depleting defects and scattering centers in PdSe_2_ channel. This engineering extends relaxation time constants from 15.6 s to 99.4 s and enables multilevel memory (>2^6^ levels) with prolonged retention (>2000 s). Leveraging dual nonlinear/stable operational modes, the physically integrated RC system achieves 91.7% (MNIST) and 93.3% (spoken digits) classification accuracy. Notably, it pioneers electrocardiogram arrhythmia detection (N, L, R, A, and V classes) with 92.3% accuracy, surpassing existing in‐memory computing approaches. By establishing a defect engineering paradigm for material‐intrinsic neuromorphic devices, this work advances energy‐efficient AI hardware for biomedical diagnostics and edge computing applications.

## Graphical abstract

A homogeneous PdSe_2_ charge‐trap memory‐based reservoir computing system is demonstrated via ultrafast photoexcitation defect engineering. Femtosecond laser‐treated devices with multilevel conductance and persistent retention are used as synapses in the readout layer, while pristine devices with short‐term plasticity serve as reservoir nodes. This physically integrated system achieves high classification accuracies, pioneers electrocardiogram detection, and advances energy‐efficient artificial intelligence hardware.

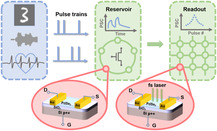

## 1 Introduction

Neuromorphic computing, a bio‐inspired computational paradigm, presents a promising solution to overcome the von Neumann bottleneck by enabling energy‐efficient artificial intelligence (AI) implementations.^[^ ^1^, ^2^, ^3^ ^]^ Owing to their atomically thin structure, scalable fabrication capabilities, and diverse physical properties, 2D materials (2DMs) have emerged as crucial building blocks for next‐generation neuromorphic electronics.^[^ ^4^, ^5^, ^6^ ^]^ Specifically, 2DMs‐based reservoir computing (RC), which integrates the advantages of low‐power hardware and lightweight neural networks capable of processing complex time‐dependent patterns with minimal training overhead, represents an ideal platform for edge AI applications, particularly in scenarios demanding rapid deployment and real‐time data processing.^[^ ^7^, ^8^, ^9^, ^10^ ^]^ An RC system operates with a typical three‐layer architecture: input layer receives information, reservoir (middle) layer functions with nonlinear adaptivity to spatiotemporal inputs, and readout layer trains to translate the reservoir output by means of linear transformation. The connections between reservoir nodes are rigid, and the network only needs to train the output layer, thus significantly minimizing the computational load.^[^ ^11^ ^]^ Researchers have demonstrated deep RC using In_2_Se_3_ synaptic devices^[^ ^12^ ^]^ and wide RC based on MoS_2_ synaptic elements,^[^ ^13^ ^]^ respectively, highlighting the rich dynamical behaviors and excellent tunability of 2DMs for advanced RC implementations.

The majority of 2DMs‐based RC systems use an analog–digital hybrid architecture.^[^ ^14^, ^15^, ^16^ ^]^ In this framework, the temporally adaptive conductance of 2D devices serves as reservoir nodes for feature extraction, while the training of the readout neural network is executed by complementary metal‐oxide‐semiconductor (CMOS) digital circuits. This mixed‐signal architecture necessitates additional analog‐to‐digital conversion during data processing, thereby introducing nontrivial energy overhead. A compelling strategy to address this challenge is to fully leverage the short‐term and long‐term plasticity of 2D synaptic devices for constructing homogeneous physically‐based RC systems,^[^ ^17^, ^18^, ^19^ ^]^ which could eliminate the need for hybrid signal conversion and further enhance energy efficiency. Only a few studies have reported all‐physical in‐sensor RC based on 2D van der Waals heterostructures.^[^ ^20^, ^21^, ^22^ ^]^ These systems utilize unstable/stable ferroelectric polarization switching under optical/electrical stimuli to realize volatile/nonvolatile memory functions. However, their multistacked architectures inherently introduce fabrication complexity and pose significant challenges for high‐density integration. Thus, realizing short‐term and long‐term memory functionalities within a compact single 2D layer rather than stacked heterostructures is highly desirable, yet such demonstrations remain scarce. This necessitates innovative design of memory retention modulation mechanisms and exploration of suitable materials. Charge‐trap dynamics mediated by defects in 2D transition‐metal dichalcogenides (TMDs) have been shown to exhibit rich synaptic behaviors.^[^ ^23^, ^24^, ^25^, ^26^ ^]^ Notably, palladium diselenide (PdSe_2_), a noble metal dichalcogenide featuring unique pentagonal puckered atomic layers,^[^ ^27^, ^28^ ^]^ demonstrates atmospheric‐sensitive bipolar conduction due to oxygen/water molecule adsorption at selenium vacancies.^[^ ^29^ ^]^ Its charge transport properties are highly sensitive to trap states modulated by external stimuli,^[^ ^30^, ^31^ ^]^ providing an ideal platform to dynamically manipulate charge trapping/detrapping kinetics for adjustable memory retention times.

In this work, through effective defect engineering by ultrafast photoexcitation with photon energy of 2.4 eV and pulse duration of 260 fs, we demonstrate a homogeneous RC system based on PdSe_2_ charge‐trap memory (CTM). The pristine CTM exhibits nonlinear fading memory suitable for reservoir state mapping, which is attributed to intrinsic interfacial trap sites that modulate fast charge detrapping. After photoexcitation, PdSe_2‐x_O_x_ nanodefects in the PdSe_2_ channel were introduced without causing structural degradation. These nanodefective sites act as electron‐depleting defects and scattering centers, significantly increasing (by a factor of 6) the relaxation time constants of the device conductance. The ultrafast photoexcited device shows multilevel potentiation/depletion states (>2^6^) and excellent retention (>2000 s, 2^4^ states), which are essential for readout training. Leveraging these dual functionalities, we implement a homogeneous RC system achieving 91.7% (MNIST) and 93.3% (spoken digits) classification accuracy. Notably, we demonstrate PdSe_2_ CTM‐based electrocardiogram (ECG) arrhythmia detection (N/L/R/A/V classes) with 92.3% accuracy, outperforming previous in‐memory computing approaches with a significantly reduced training cost. This work establishes a defect‐engineering paradigm for developing material‐intrinsic neuromorphic devices, significantly advancing the realization of energy‐efficient AI hardware.

## 2 Results and Discussion

### 2.1 Device Characteristics

**Figure 1:**
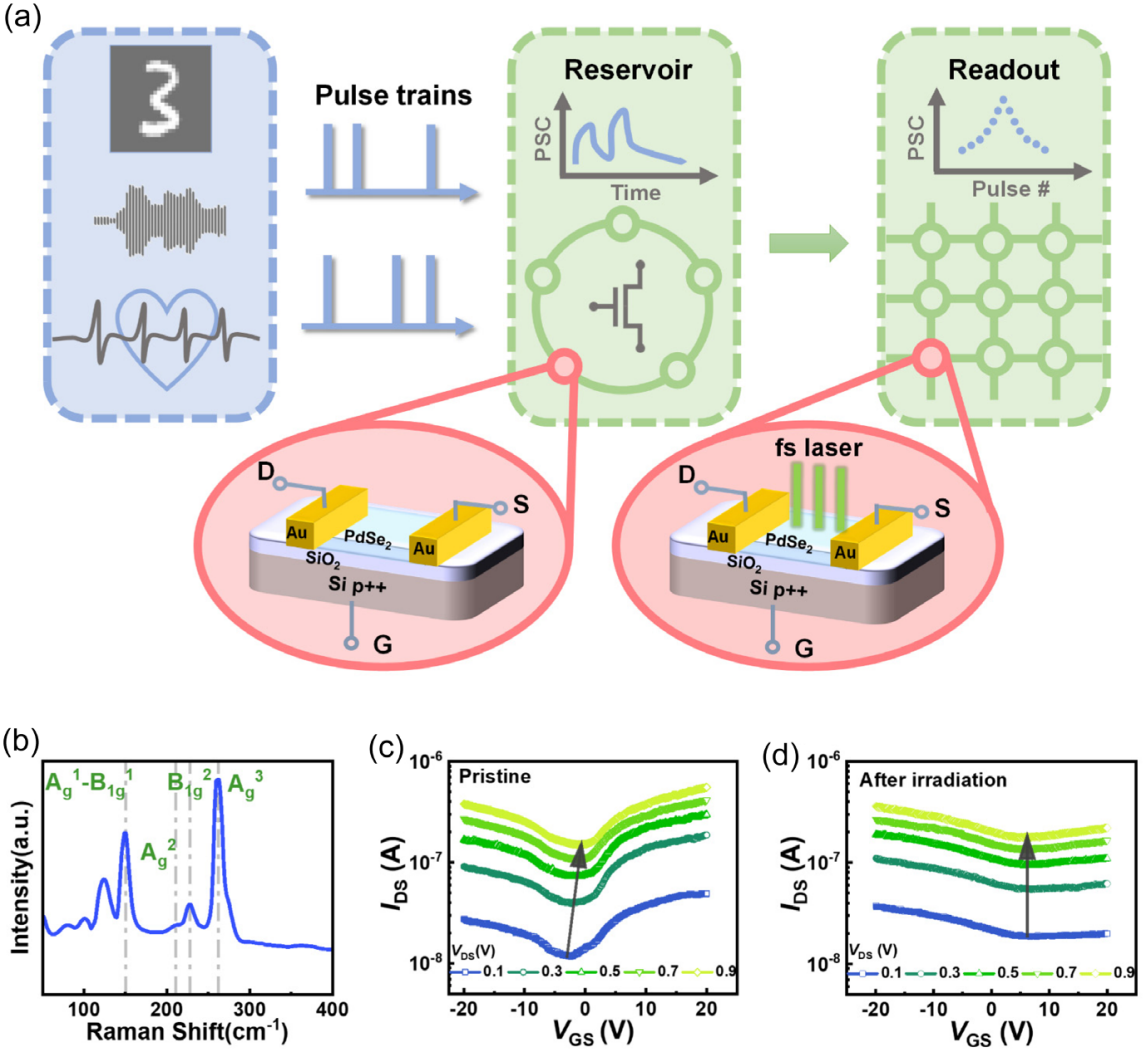
Schematic diagram of the physically integrated RC system and electronic properties of PdSe_2_ CTM. a) Schematic diagram of PdSe_2_ CTM‐based RC system for various information processing; the pristine devices with volatile memory serve as adaptive reservoirs while the devices subjected to ultrafast photoexcitation with long‐term memory construct a single‐layer perception readout. b) Raman spectrum of the exfoliated PdSe_2_ flake; four characteristic peaks corresponding to A_g_ and B_1g_ vibrational modes are observed. Transfer curves of the device c) before and d) after fs laser irradiation.

Schematic diagram of the physically integrated RC system based on PdSe_2_ CTM devices is shown in **Figure** **1**a. An optical microscopy image of the PdSe_2_ CTM is presented in Figure S1, Supporting Information. The conducting channel dimensions is 5.2 μm in width and 5.0 μm in length. The atomic flatness of the exfoliated flake was verified by atomic force microscopy (AFM), with AFM analysis confirming a thickness of ≈5 nm (Figure S1 inset, Supporting Information). Raman spectrum of the PdSe_2_ 2D flake exhibits four distinct vibrational modes at 149.8, 211.0, 227.6 and 262.3 cm^−1^ respectively (Figure 1b), which are consistent with the characteristic A_g_ and B_1g_ phonon modes reported for multilayered PdSe_2_.^[^ ^32^ ^]^

The transfer curves of the pristine device under different drain‐source voltages (*V*_DS_) exhibit typical ambipolar behavior (Figure 1c). The minimum conducting points (*V*_GS_^min^) show a slight deviation from zero gate bias (*V*_GS_) toward negative values, suggesting intrinsic asymmetry of the electron‐hole conduction.^[^ ^33^ ^]^ Increasing *V*_DS_ elevates channel currents while maintaining the ambipolar characteristics. The dependence of *V*_GS_^min^ on different *V*_DS_ is depicted in Figure S2a, Supporting Information. The continuous shift of *V*_GS_^min^ from −2 to 0 V with increasing *V*_DS_ suggests lateral electric field‐modulated charge trapping at PdSe_2_/SiO_2_ interfacial states.^[^ ^34^ ^]^ After fs laser irradiation, a significant suppression of the n‐type branch emerges (Figure 1d). The resultant hole‐dominant transport behavior, accompanied by a positive *V*_GS_^min^ shift, indicates device transformation into a *p*‐type depletion‐mode transistor. Notably, the *V*_GS_^min^ remains invariant with *V*_DS_ variations (Figure S2b, Supporting Information), implying effective mitigation of interface trap‐mediated effects.

Doping effects induced by ultrafast laser processing have been widely observed in graphene,^[^ ^35^ ^]^ TMDs,^[^ ^36^ ^]^ and van der Waals heterostructures.^[^ ^37^ ^]^ Photochemical oxidation is generally considered the dominant mechanism.^[^ ^38^ ^]^ The fs laser‐induced electron thermalization process preferentially generates selenium vacancies in PdSe_2_, which act as adsorption sites for ambient oxygen molecules through combined physisorption and chemisorption pathways. Oxygen's high electronegativity induces substantial electron transfer from PdSe_2_, resulting in pronounced electron depletion within the conduction channel. It should be noted that fs laser parameters (pulse width, power density, and repetition rate) were carefully adjusted to well below the ablation threshold of PdSe_2_. No evidence for surface oxide formation or laser‐induced exfoliation was observed by Raman spectroscopy and AFM analysis (Figure S3 and S4, Supporting Information). Detailed analysis of the fs laser‐induced localized modification mechanism will be systematically presented in the following section.

The field‐effect mobility *μ*_FE_ of PdSe_2_ devices was quantitatively evaluated before and after fs laser treatment using the standard expression.^[^ ^39^ ^]^

(1) $$\mu_{FE}\;=\;\frac{\partial I_{DS}}{\partial V_{GS}}\;\times \;\frac{L}{W}\;\times \;\frac{1}{C_{ox}V_{DS}}$$

where *L* and *W* represent the channel length and width, respectively, and *C*_ox_ is the gate capacitance per unit area. For 300 nm SiO_2_, a dielectric capacitance of 11.5 nFcm^−2^ was used.^[^ ^30^ ^]^ Linear fittings of transfer curves measured at *V*_DS_ = 0.3 V are shown in Figure S5, Supporting Information. The pristine device exhibits hole and electron mobilities of 0.20 cm^2^V^−1^s^−1^ and 0.62 cm^2^V^−1^s^−1^, respectively. A ≈94% reduction in electron field‐effect mobility (0.04 cm^2^V^−1^s^−1^) is obtained for the laser‐treated device, while the hole mobility remains almost unaffected (0.21 cm^2^V^−1^s^−1^). In addition, the dependence of output current on *V*_DS_ (Figure S6, Supporting Information) shows better linearity in the laser‐treated device, suggesting reduced Schottky barrier between PdSe_2_ and metal contacts.

### 2.2 Memory Loss Modulation Through Defect Engineering

**Figure 2:**
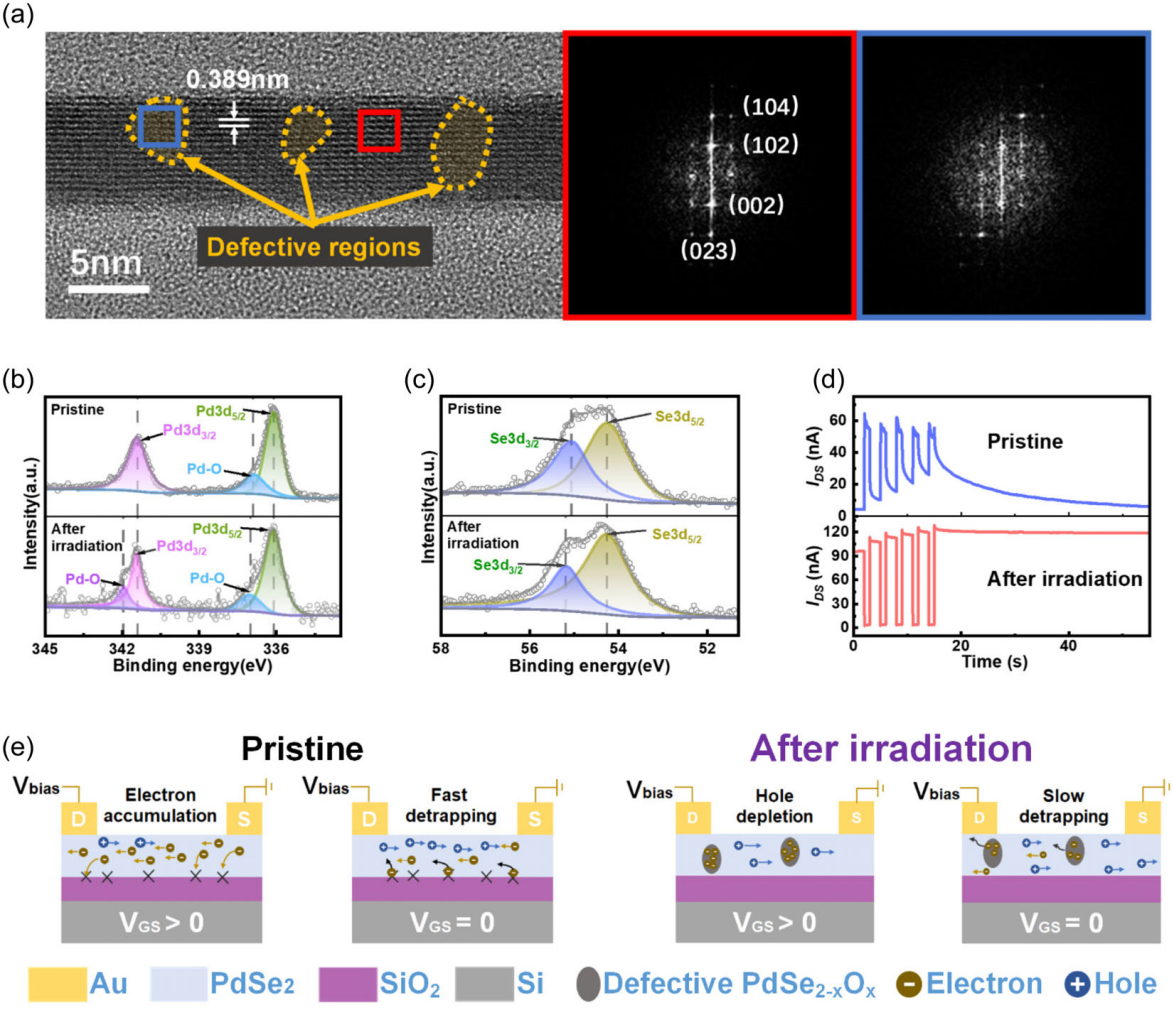
Defect engineering in PdSe_2_ by fs laser irradiation. a) Cross‐sectional HRTEM image and FFT patterns of fs laser‐treated PdSe_2_; defective regions are demarcated by dotted curves with semitransparent yellow fill (20% opacity) for clear visualization. High‐resolution XPS spectra of b) pristine and c) fs laser‐excited PdSe_2_. d) PSC of devices with and without ultrafast photoexcitation subjected to five consecutive gate pulses (+15 V, 1s). e) Schematic illustration of short‐term and long‐term memory in PdSe_2_ CTM through the modulation of charge trapping/detrapping processes by interfacial trap sites and PdSe_2‐x_O_x_ defective nanoregions.

In contrast to continuous‐wave lasers inducing significant thermal effects in 2DMs, fs laser processing enables precise modification of the local structure and composition in layered materials with minimal thermal dissipation.^[^ ^40^ ^]^ To elucidate the fs laser‐induced structural evolution, cross‐sectional high‐resolution transmission electron microscopy (HRTEM) analysis was conducted. **Figure** **2**a reveals the layered atomic structure of laser‐treated PdSe_2_. The measured interlayer spacing of ≈0.389 nm matches the (002) lattice plane of the *Pbca* orthorhombic structure. Notably, a number of defect‐rich nanoregions (outlined by semitransparent yellow shading) are embedded within the crystalline matrix, as revealed by fast Fourier transform (FFT) patterns. It is observed that defective nanoregions are not confined to the surface area; instead, a certain number of defective zones are distributed throughout the entire layer. This is evidenced by high‐resolution images of different regions within the PdSe_2_ layer, as presented in Figure S7, Supporting Information. Complementary energy‐dispersive X‐ray spectroscopy (EDS) mapping (Figure S8, Supporting Information) confirms oxygen incorporation in defect zones. Quantitative analysis presents the atomic percentage of Pd, Se, and O are 10.01%, 18.83%, and 5.66% respectively, indicating deficient Se atoms under the excitation of fs laser. These observations align with established mechanisms of chalcogen vacancy formation under ultrafast laser excitation.^[^ ^41^ ^]^ It is reasonable to hypothesize that the extremely high instantaneous energy of fs pulsed lasers can induce a large number of Se vacancies in ultrathin PdSe_2_ films. These vacancies, in turn, facilitate the diffusion of O_2_ molecules into the PdSe_2_ crystal lattice, ultimately leading to the formation of PdSe_2‐x_O_x_ defective regions.

X‐ray photoelectron spectroscopy (XPS) was performed to probe chemical states of Pd and Se in both pristine and fs laser‐irradiated PdSe_2_, as shown in Figure 2b,c. High‐resolution XPS spectra were fitted with Gaussian peaks. The pristine PdSe_2_, Pd 3 d peaks are located at 336.1 eV (3d_5/2_) and 341.4 eV (3d_3/2_), which is in accordance with previous reports.^[^ ^42^ ^]^ Note that the small peak at 336.9 eV could be assigned to the chemical bonds between Pd and atmospheric oxygen in place of intrinsic Se vacancies. After fs laser treatment, an additional peak at higher energy (341.9 eV) is observed, which is attributed to the ultrafast laser‐initiated Pd—O bond formation. Moreover, the 336.9 eV peak intensity increases, suggesting larger amount of Pd bonded to oxygen with the help of fs laser excitation. The binding energy of Se 3 d is measured to be 54.3 eV (3d_5/2_) and 55.1 eV (3d_3/2_) for the pristine sample. A 0.1 eV shift in Se 3d_3/2_ peak position (55.2 eV) is observed after fs laser treatment, which is resulted from modified bonding environment with increased number of oxygens. The obtained results confirm oxygen chemisorption at selenium vacancy sites, forming PdSe_2‐x_O_x_ phases that dominate charge trapping dynamics.

The engineered defects fundamentally alter synaptic responses in PdSe_2_ CTM devices. Figure 2 d compares the transient currents under +15 V gate pulses (1 s duration, 5 cycles). The pristine device exhibits a postsynaptic current (PSC) that increases during the gate pulse and rapidly decays to baseline within tens of seconds. In contrast, the defect‐modified device shows decreased conductance during pulse application, followed by stable current retention, indicating nonvolatile memory characteristics. Through double‐exponential fitting of the current decay curves after pulse termination, two relaxation time constants (*τ*_1_ and *τ*_2_) were extracted (Figure S9, Supporting Information). The significant enhancement of *τ*_2_ from 15.6 s to 99.4 s in defect‐modified device implies slower charge detrapping dynamics, which is attributed to the presence of PdSe_2‐x_O_x_ defect complexes.

Based on comprehensive material characterization and meticulous electrical measurements, we put forward a well‐founded explanation for the charge trap dynamics of PdSe_2_ CTM, as depicted in Figure 2e. In pristine devices, electron accumulation under positive gate bias increases channel conductance. Electrons are temporarily trapped at interfacial states but rapidly detrap after bias removal. In contrast, for the devices with PdSe_2‐x_O_x_ defect nanoregions, a substantial number of electrons are transferred and trapped in the defective zones due to the high electronegativity of oxygen. The resultant *p*‐type conduction causes the depletion of holes under a positive presynaptic input, leading to a decrease in the channel conductance. Because of the significantly suppressed mobility, electrons trapped in the nanodefective regions escape at a rather slow pace, enabling nonvolatile PSC retention.

### 2.3 Reservoir Devices and Nonvolatile Artificial Synapses

**Figure 3:**
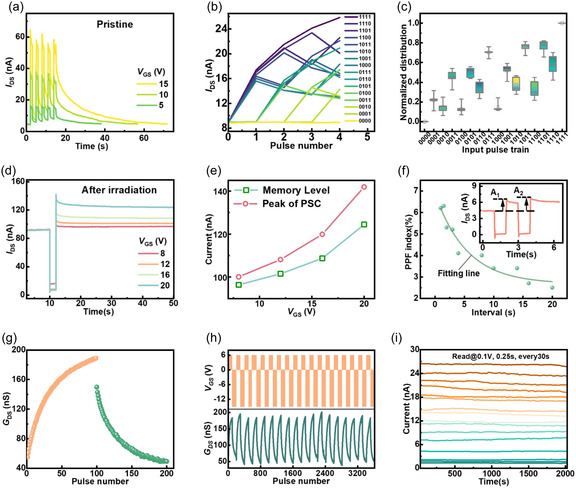
Short‐term and long‐term memory of PdSe_2_ CTM for reservoir mapping and readout perception, respectively. a) PSC of the pristine device subjected to varied *V*_GS_ pulse amplitudes (5, 10, and 15 V, 1 s). b) Adaptive current response of the pristine device to 16 different input pulse trains for four‐bit signal separation. c) Distribution of normalized response currents of five fabricated devices to four‐bit sequential inputs. d) PSC of fs laser‐treated device under different pulse amplitudes (8, 12, 16, and 20 V, 1 s). e) Measured PSC peaks and memory levels of fs laser‐treated device as a function of pulse amplitudes (*V*_GS_). f) PPF index as a function of pulse intervals. g) LTP/LTD characteristics of 200 consecutive pulses. h) Continuous LTP/LTD cycles of PdSe_2_ CTM after fs laser irradiation. i) Retention of multilevel currents (2^4^) for 2000 s.

The short‐term plasticity of the as‐fabricated device under *V*_GS_ pulses with amplitudes of 5, 10, and 15 V is shown in **Figure** **3**a. Five consecutive pulses with a 1 s width were applied. Larger *V*_GS_ amplitudes generated higher PSC, yet the memory level, which is defined as the steady‐state current over time, quickly decreases to baseline within tens of seconds, independent of *V*_GS_. The current response of the device under a smaller *V*_*GS*_ is shown in Figure S10 of the Supporting Information. The PSC exhibits a steep drop from the peak; meanwhile, minimal memory effect is observed when *V*_*GS*_ is reduced to 1 V.

By exploiting the dynamic conductance loss and nonlinear short‐term memory of pristine PdSe_2_‐based CTM as physical reservoirs, temporal signal encoding was achieved. Four‐bit pulse trains were applied as temporal inputs, where pulse voltages of 0 V and +15 V with a fixed 1 s width represented binary states “0” and “1,” respectively. A 0.5 s pulse interval was used to enhance inter‐pulse correlation. The response currents to pulse trains ranging from “0000” to “1111” are presented in Figure S11, Supporting Information. Current states corresponding to 16 distinct pulse sequences were recorded 1 s after the termination of each input signal. As shown in Figure 3b, the 16 final current outputs demonstrate clear separability, attributable to dynamic carrier relaxation from interfacial charge traps in the pristine PdSe_2_ CTM. To ensure reliable mapping of repeated input signals to consistent reservoir states with minimal variation, we evaluated the device consistency using two representative pulse sequences (“1100” and “1011”) over 10 cycles (Figure S12, Supporting Information). The relative standard deviation (RSD) of current values for each pulse remained below 2%, confirming the stability of our reservoir device.

Device‐to‐device variations were systematically assessed by measuring current responses to four‐bit sequential inputs across four additional fabricated devices (Figure S13, Supporting Information). Notably, the inherent thickness variations in mechanically exfoliated PdSe_2_ flakes resulted in baseline current differences. To enable comparative analysis, all 16 current states were normalized and the reservoir state distributions were statistically evaluated. Figure 3c demonstrates well‐separated clusters for different inputs, highlighting excellent consistency among pristine devices when used as reservoir nodes for parallel signal processing.

The nonvolatile PSC observed in fs laser‐treated PdSe_2_ CTM exhibits marked contrast with the volatile behavior of pristine device, as demonstrated in Figure 3 d. Increasing the *V*_GS_ not only enhances the current peak but also elevates memory retention levels. Transient output currents under *V*_GS_ pulses with varied pulse number (N) and pulse duration (tp) were further investigated (Figure S14, Supporting Information). Figure 3e demonstrates the spiking‐strength‐dependent plasticity through PSC peaks and memory levels under varying *V*_GS_, a critical feature for emulating biological learning and memory processes. Moreover, paired‐pulse facilitation (PPF) is also examined through varying the pulse intervals (Δ*t*) between two input pulses, as shown in Figure 3f. The PPF index ($\frac{A_{2}-A_{1}}{A_{1}}\times 100\%$) shows an exponential increase with reduced Δ*t*, which is a good emulation of dynamic modulation in synapses for temporal filtering of neural signals.

To validate synaptic programmability of defect‐engineered devices for readout layer of RC, an initial investigation was conducted on their long‐term potentiation and depression (LTP/LTD) characteristics. Figure 3g shows the continuous conductance modulation through sequential application of 100 positive (+6 V, 1 s) and 100 negative (−15 V, 1s) *V*_GS_ pulses. Similar results can be obtained in other four devices (Figure S15, Supporting Information). Cycling stability was verified through 18 consecutive LTP/LTD operations consisting 3600 pulses, as shown in Figure 3h. Moreover, Figure 3i shows the multistate retention characteristics of the device; 16 distinct conductance levels (2^4^) were recorded over 2000 s, demonstrating retention stability with clear state‐to‐state separation. Eight distinct current states were selected, and the peaks of the PSC and memory levels are summarized in Figure S16, Supporting Information. A quantitative evaluation of variability across 67 read‐level operations—using mean values (μ), standard deviations (σ), and RSDs—is presented in Table S1, Supporting Information. The results reveal remarkably stable performance, with all RSD values less than 0.022, demonstrating negligible current drift over the 2000 s retention time.

### 2.4 All‐Hardware RC for Image and Spoken Digits Recognition

The PdSe_2_ CTM can be effectively modulated to exhibit both remarkable volatile and outstanding nonvolatile PSC, which enables the construction of an all‐hardware RC framework. Devices dominated by interfacial traps, serving as physical reservoir nodes, were used to map the input signals to higher‐dimensional reservoir states. Meanwhile, devices with nanodefective regions, acting as synapses in the output layer, were trained for analog readout.

**Figure 4:**
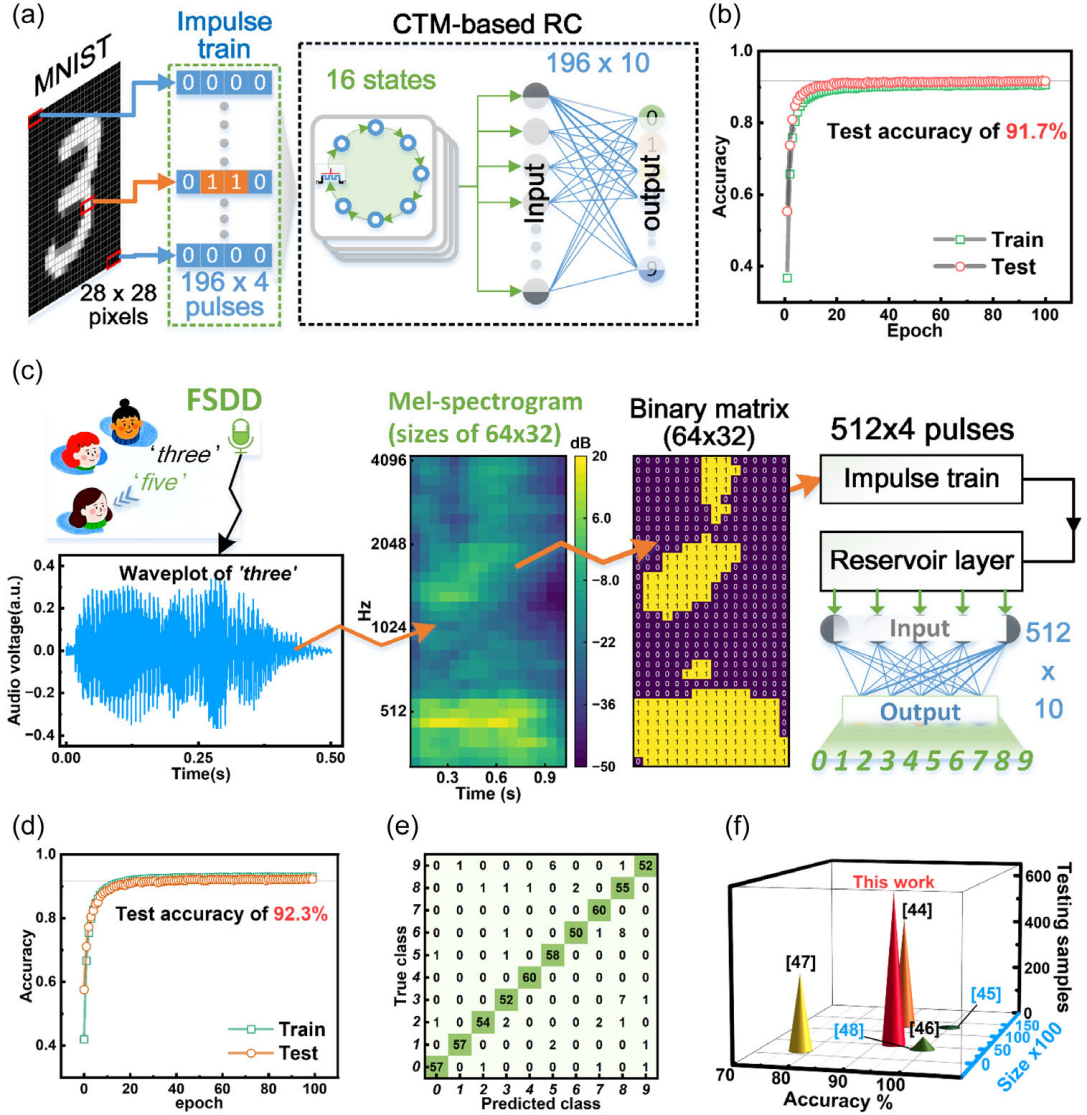
Image and spoken digits recognition implemented by PdSe2 CTM‐based all‐hardware RC system. a) Schematic diagram of the training process on handwritten digit image. The image sizes are 28 × 28 pixels and the dynamic reservoir layer has 16 physical reservoir states. Meanwhile, the weight update of the readout layer is done in 128 discrete values mapped by the device conductance. b) Training and testing accuracy on MINIST as a function of epoch. c) Schematic of the FSDD‐based speech recognition process. The voice .wav file is directly transformed by Mel‐spectrum. The set parameters of the Mel transform are 64 banks of filters and the FFT length is 2048 sample points. The hop length is 1/4 of the FFT length. d) Training and testing accuracy on FSDD as a function of epoch. e) Confusion matrix results on 600 test samples (60 per class). f) Comparison of key parameters of network performance between this study and other works, refs. [45] and [48] did not present the results of the test set, so the number of testing samples was zero.

The RC system was first validated on a reduced MNIST dataset containing 10 000 training images (1000 per digit class) and 2000 test images (200 per class). Each 28 × 28‐pixel grayscale image was preprocessed into a 196 × 4 binary matrix and converted to a temporal pulse sequence. These sequences were input to the CTM‐based RC network with a reservoir layer comprising 16 pristine PdSe_2_ devices. The readout layer used fs laser‐irradiated PdSe_2_ CTMs, forming a fully connected network with 196 × 10 synaptic weights (**Figure** **4**a). Weight updates utilized 64 discrete resistance states per polarity (128 total states), mapped to corresponding positive/negative weight ranges. Through mixed hardware‐software training, the system achieved 91.7% test accuracy (Figure 4b), with per‐class recognition metrics detailed in Figure S17, Supporting Information. Notably, the framework maintained competitive performance (89.8% accuracy) on the full MNIST dataset (60 000 training/10 000 test samples (Figure S18 and S19, Supporting Information). Implementation specifics of the hybrid training strategy are provided in Note S1, Supporting Information.

The RC architecture was further evaluated on the Free Spoken Digit Dataset (FSDD),^[^ ^43^ ^]^ containing 3000 English digit recordings (0–9) from six speakers. Voice recordings (8 kHz .wav format) were trimmed to remove leading/trailing silence. Each voice wav file was first converted to a 64‐channel Mel‐spectrogram, which is a nonlinear spectrogram based on human auditory perception. The current corresponding Mel‐frequency matrix has a spectral size of 64 × 32 and consists of decibel values in the frequency and time domains. After a decibel threshold division, it can be transformed into a binary matrix, and then divided into four sites to form a pulse sequence of 512 × 4. The conversion process from speech waveform to pulse sequence is shown in Figure 4c. The readout layer size of the corresponding RC system is 512 × 10. Each category of speech digits in FSDD was randomly divided into training and test sets in a ratio of 8:2. The RC system achieved 93.3% accuracy on the test set containing 600 records, as shown in Figure 4 d. The confusion matrix (Figure 4e) and comparative analysis with existing implementations^[^ ^44^, ^45^, ^46^, ^47^, ^48^ ^]^ (Figure 4f) highlight superior inference capability. Remarkably, the compact readout architecture (5120 trainable parameters) for a large number of test samples demonstrates strong generalization while minimizing power overhead, which is a critical advantage for edge computing applications.

### 2.5 ECG Classification

Edge AI devices have emerged as promising platforms for medical diagnostics, enabling high‐accuracy screening with minimal hardware resource consumption and energy expenditure. In this context, we demonstrate the implementation of CTM‐based all‐hardware RC system for energy‐efficient arrhythmia classification.

**Figure 5:**
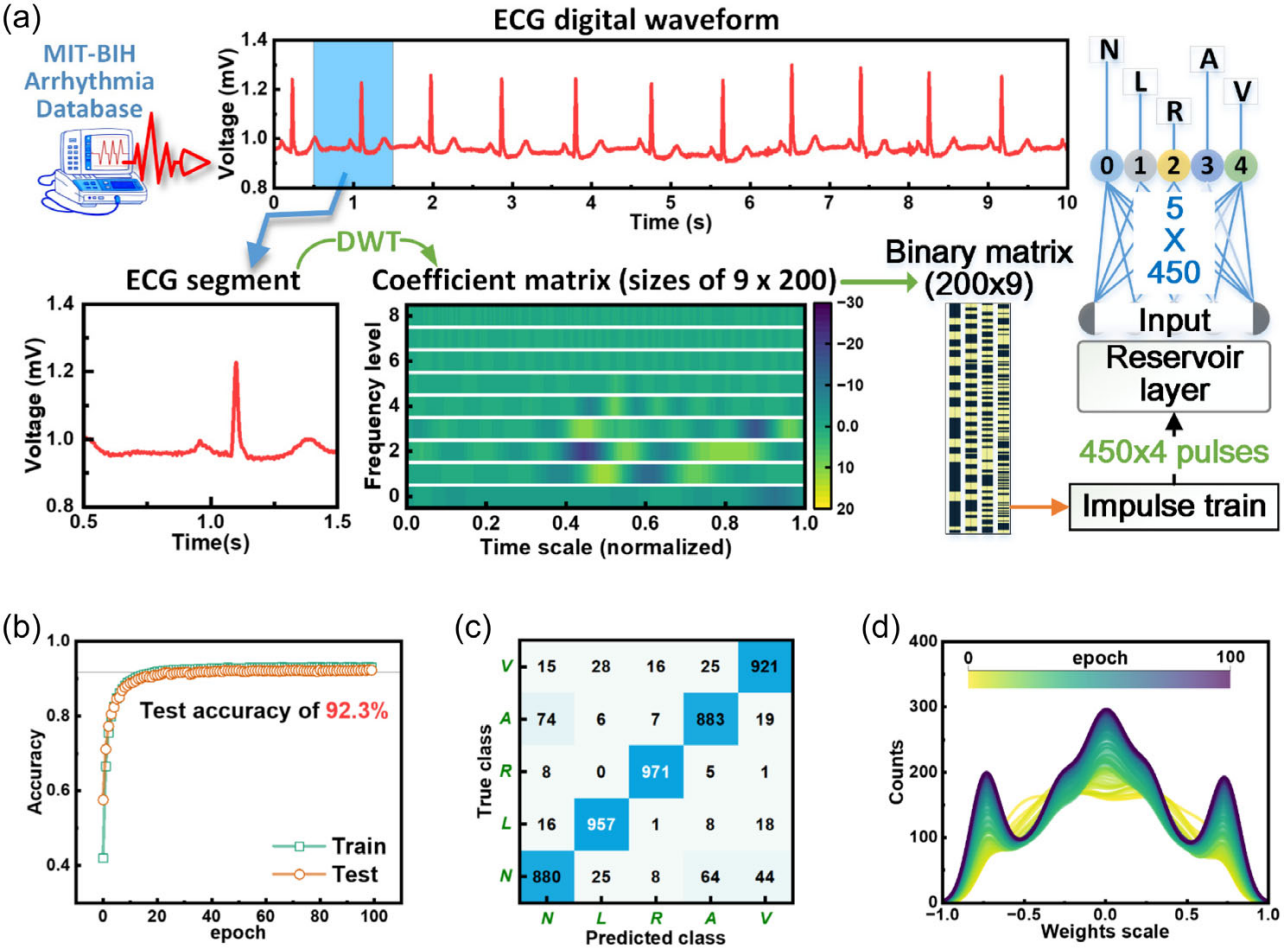
a) Schematic diagram of the pre‐processing flow for ECG recording for arrhythmia classification. The used wavelet basis function is “db6” with 9 frequency levels in DWT for ECG segment. The initial coefficient matrix obtained by DWT has different time sampling at different frequency levels, thus it is uniformly resampled to 200 according to the most time sampling points. b) The training and testing accuracy as a function of epoch. c) Confusion matrix results based on 5000 test samples. d) The normalized weight value distribution of the readout layer changes with epoch from 0 to 100 during training.

The network performance was evaluated using the MIT‐BIH arrhythmia dataset containing 48 30‐min ECG recordings from 47 subjects, annotated with five arrhythmia classes (N, L, R, A, and V). Signals were digitized at 360 Hz and segmented into 25 000 1‐s heartbeat‐aligned samples (5000 per class). To address signal sparsity and feature preservation challenges, we implemented a discrete wavelet transform (DWT)‐based preprocessing pipeline, as shown in **Figure** **5**a. Each ECG segment was converted to a 9 × 200 coefficient matrix capturing time‐frequency characteristics, followed by threshold binarization and restructuring into 450 × 4 pulse trains. The dataset was partitioned into 80% training and 20% testing subsets with stratified sampling.

As shown in Figure 5b, the hybrid hardware‐software RC system achieved 92.3% overall inference accuracy on 5000 test samples, with class‐specific accuracies ranging from 88 to 97.1% (Figure 5c). This performance surpasses the 91% accuracy reported for a 2000‐sample test set using multilayer perceptron networks.^[^ ^49^ ^]^ Notably, the number of parameters requiring training in the readout layer of the RC system is only 2250 (450 × 5), which is considerably lower than that of conventional ANN networks reported in the literature (150 000).^[^ ^49^ ^]^ Figure 5d illustrates the dynamic distribution of synaptic weights during training. The progressive convergence of weight values demonstrates efficient network optimization, suggesting practical applicability for medical diagnostic systems with enhanced computing efficiency.

## 3 Conclusions

In this work, we demonstrate a homogeneous hardware RC system based on defect‐engineered 2D PdSe_2_ CTM. Ultrafast photoexcitation‐induced Se vacancies initiate oxygen chemisorption to form PdSe_2‐x_O_x_ nanodefects. Unlike interfacial traps in pristine CTM that enable rapid charge detrapping and short‐term characteristics, these engineered defects serve as effective scattering centers that suppress electron mobility, leading to prolonged charge detrapping processes with a remarkable ≈6.4‐fold enhancement of time relaxation constant in PSC. As a result, long‐term memory retention is achieved. The dynamic memory properties of pristine device are exploited to generate 16 distinct reservoir states for 4‐bit sequential inputs, while fs laser‐treated devices exhibit multilevel conductance (>2^6^ states) with persistent retention (>2000 s), suitable for readout network training. Our PdSe_2_ CTM‐based homogeneous RC framework achieves high recognition accuracies of 91.7% (MNIST dataset) and 93.3% (FSDD dataset). Furthermore, it successfully accomplishes medical diagnostics for ECG classification, attaining 92.3% accuracy. The demonstrated synergy between controlled defect engineering and neuromorphic computing principles opens new avenues for material‐driven neuromorphic hardware design.

## 4 Experimental Section

4.1

4.1.1

##### Device Fabrication

PdSe_2_ bulk crystals were purchased from 6Carbon Technology (Shenzhen, China). PdSe_2_ nanosheets were mechanically exfoliated onto a polydimethylsiloxane (PDMS) film and transferred onto a Si substrate capped with a 300 nm‐thick SiO_2_ layer using a dry transfer method under optical microscopy guidance. Source and drain electrodes were patterned by ultraviolet (UV) photolithography, followed by thermal evaporation of Cr/Au (5/30 nm) layers. The substrate was subsequently immersed in acetone for 30 min during the lift‐off process, rinsed with deionized water, and dried under nitrogen flow to complete the memtransistor fabrication. The channel material was laser‐irradiated in ambient conditions using a Pharos‐10 W femtosecond laser system (Light Conversion) with a wavelength of 515 nm, energy density of 2.5 mJ cm^−2^, repetition rate of 1 MHz, pulse duration of 260 fs, and a focused spot size of ≈4.5 μm; the irradiation time to the PdSe_2_ device is ≈600 ms.

##### Material Characterization and Electrical Measurement

Raman spectroscopy was performed using a Horiba LabRAM HR Evolution Visible‐NIR system equipped with a 532 nm excitation laser. AFM (Bruker Multimode 8) was used to determine the thickness of PdSe_2_ nanosheets. XPS (AXIS SUPRA+, Kratos Analytical) was conducted to analyze the elemental composition of PdSe_2_ before and after laser treatment. The microstructural evolution and elemental distribution of laser‐irradiated PdSe_2_ were investigated by transmission electron microscopy (TEM, JEOL JEM‐2100 F). Electrical measurements, including DC current–voltage (I–V) curves and pulsed switching characteristics, were acquired using a Keithley 2636B SourceMeter under ambient conditions.

##### Details of the Dataset

1) MNIST dataset: MNIST is short for Modified National Institute of Standards and Technology database. The MNIST dataset is a large collection of handwritten digits, often used for benchmarking machine learning algorithms in the field of image processing. It contains 60 000 training examples and 10 000 test examples of 28 × 28 pixel grayscale images representing single digits between 0 and 9. 2) Reduced MNIST dataset: it is a reduced version of the MNIST handwritten digits where the training images are 10 000 images, 1000 for each class, and the test images are 2000 with 200 images for each class, and each image is of dimensions (28 × 28 pixels). 3) FSDD: it contains recordings of spoken digits in .wav files at 8 kHz. The dataset recordings have been trimmed at the beginning and end so that they have near‐minimal silence. This dataset contains 50 recordings for every 10 digits per every 6 speakers. This dataset in total contains 3000 recordings in English pronunciations. 4) MIT‐BIH arrhythmia dataset: it contains 48 half‐hour excerpts of two‐channel ambulatory ECG recordings, obtained from 47 subjects studied by the BIH Arrhythmia Laboratory between 1975 and 1979. The recordings were digitized at 360 samples per second per channel with 11‐bit resolution over a 10‐mV range. Two or more cardiologists independently annotated each record; disagreements were resolved to obtain the computer‐readable reference annotations for each beat (≈110 000 annotations in all) included with the database. 5) **Table** **1** presents a comparison of the parameter sizes of the RC system across different tasks.

**Table 1:** Comparison of the parameter sizes of different tasks.

Datasets	Training samples	Test samples	RC nodes	Readout sizes
MNIST dataset	60 000	10 000	196	196 × 10
Reduced MNIST dataset	10 000	2000	196	196 × 10
FSDD	2400	600	512	512 × 10
MIT‐BIH arrhythmia dataset	25 000	5000	450	450 × 5

## Supporting Information

Supporting Information is available from the Wiley Online Library or from the author

## Conflict of Interest

The authors declare no conflict of interest.

## Supporting information

Supplementary Material

## Data Availability

The data that support the findings of this study are available from the corresponding author upon reasonable request.
