# Supplementary material for: Charge‐Trap Memory with Engineered Temporal Dynamics for Physically Integrated Reservoir Computing
Source: Small Sci. 2025 Sep 29;5(12):e202500356. doi: 10.1002/smsc.202500356 (PMC12697835; doi:10.1002/smsc.202500356)
Supplement: Supplementary file 1 — Supplementary Material [file SMSC-5-e202500356-s001.pdf]

Supporting Information

Charge-trap memory with engineered temporal dynamics for physically integrated reservoir computing

Mengfan Wu[1, ⊥], Ziqi Chen[2, ⊥], Niannian Yu[1, 2 *], Leyao Li[1], Xinhao Zhang[1], Xinyi Wan[1], Yi Zheng[2], Shuaishuai Xu[4], Yang Liu[1], Jiawei Peng[1], Yao Wang[1], Junhui Yuan[1], Jiafu Wang[1], Xuewen Wang[3, *]

1 School of Physics and Mechanics, Wuhan University of Technology, Wuhan 430070, China;

2 School of Artificial Intelligence, Jianghan University, Wuhan 430056, China;

3 Center of Femtosecond Laser Manufacturing for Advanced Materials and Devices, State Key Laboratory of Advanced Technology for Materials Synthesis and Processing, Wuhan University of Technology, Wuhan 430070, China;

4 International school of Materials Science and Engineering (school of Materials and Microelectronics), Wuhan University of Technology, Wuhan 430070, China.

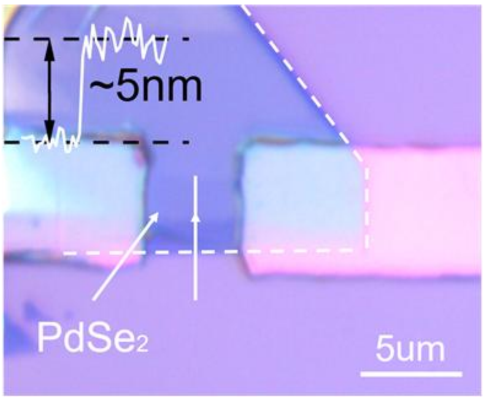

Figure S1. Optical microscopy image of the as-fabricated $PdSe_2$ CTM device. The thickness of the flake is measured to be ~5 nm by atomic force microscopy.

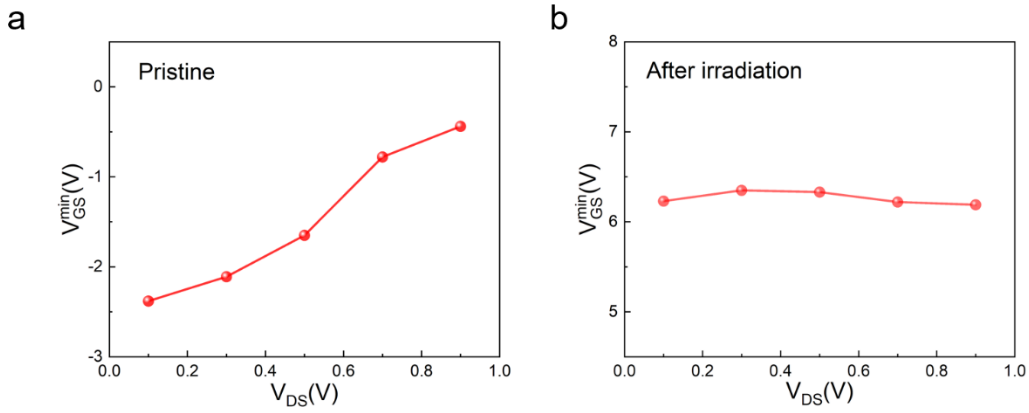

Figure S2. Minimum conducting point (V_{GS}^{min}) of transfer curves as a function of drain-source voltage (V_{DS}) for the device (a) before and (b) after fs laser treatment.

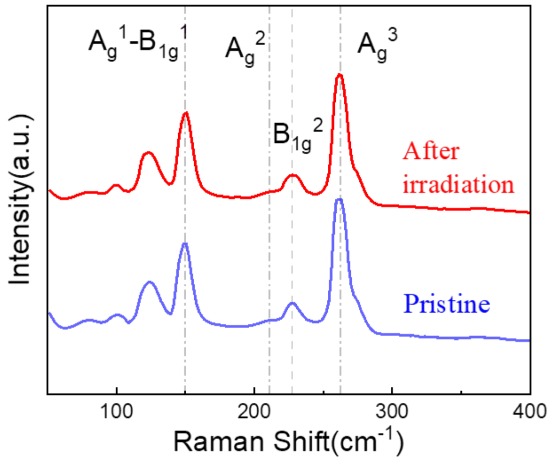

Figure S3. Raman spectra of $PdSe_2$ flake before and after fs laser irradiation. No variation in the peak positions is observed after laser treatment.

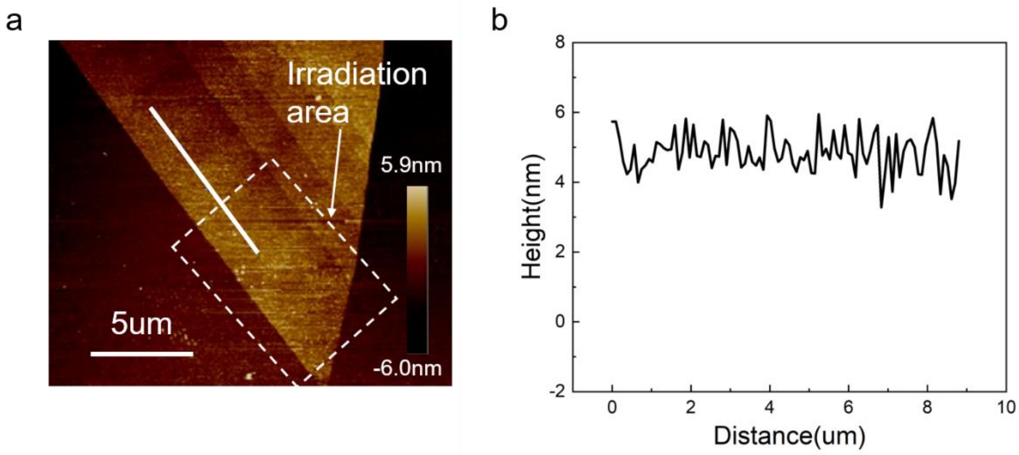

Figure S4. Morphology image of a $PdSe_2$ nanoflake obtained by AFM measurement, the area treated by fs laser is marked with a white frame. No difference in thickness between the pristine and laser-treated area is observed.

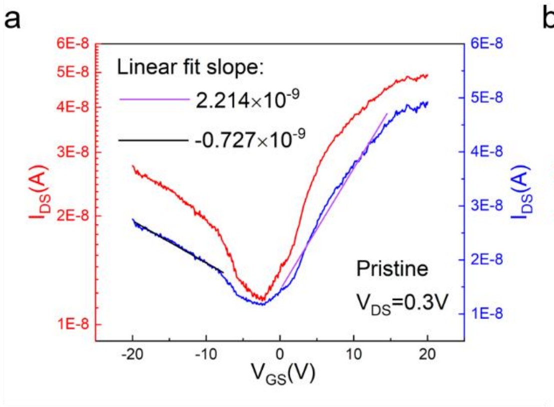

Figure S5. Linear fittings of the transfer curves for $PdSe_2$ device (a) before and (b) after fs laser irradiation.

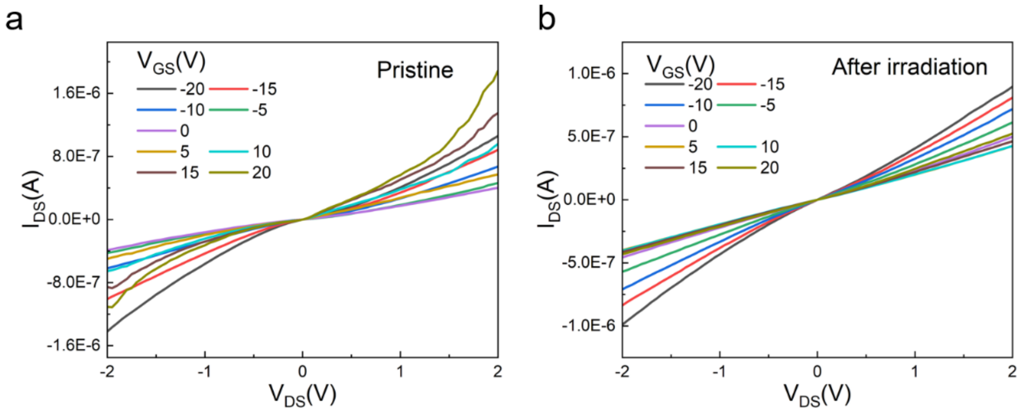

Figure S6. Output current of $PdSe_2$ device with varied V_{GS} (a) before and (b) after fs laser irradiation.

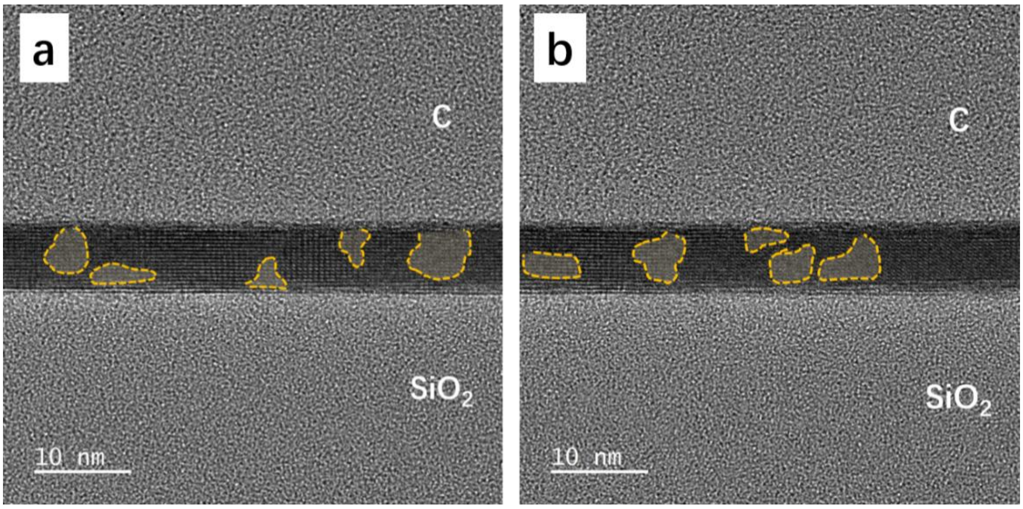

Figure S7. High-resolution TEM images of defective $PdSe_{2-x}O_x$ generated in different sites of the device

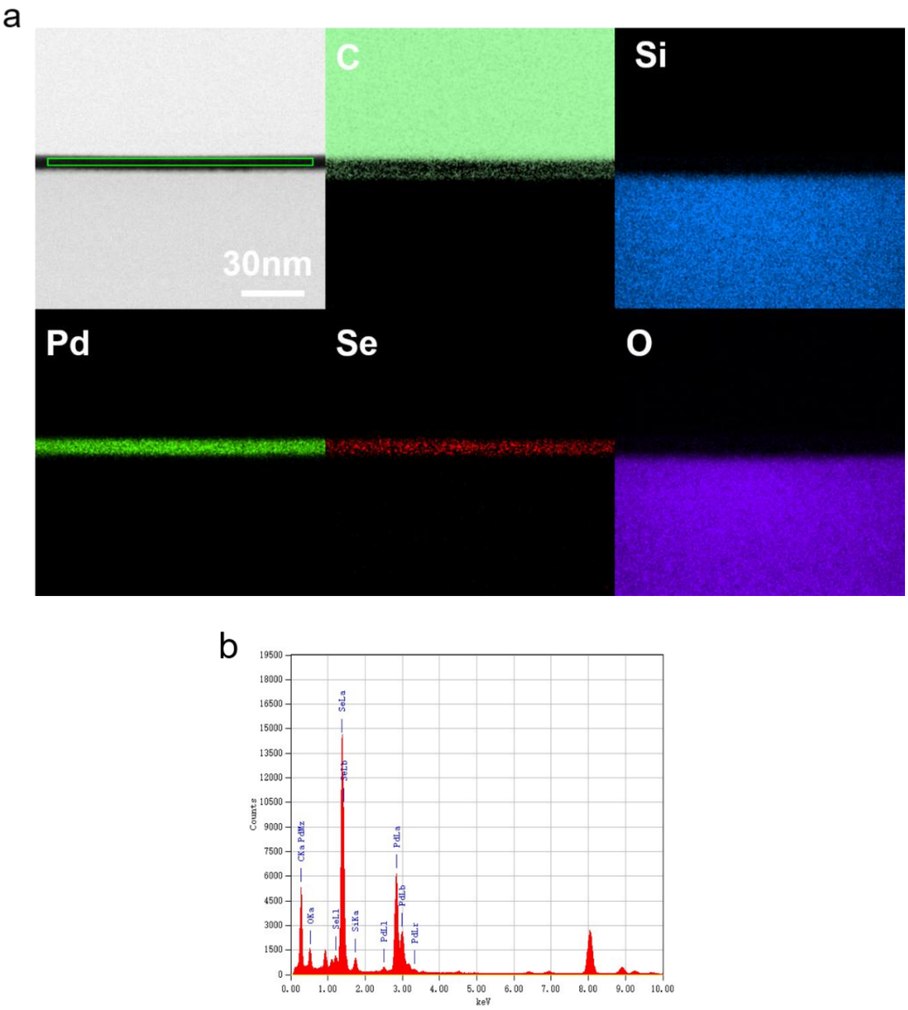

c

Thin Film Standardless Standardless Quantitative Analysis

Fitting Coefficient : 0.2721

Element		(keV)	Mass%	Counts	Sigma	Atom%	Compound	Mass%	Cation	K
C K		0.277	22.18	27072.30	0.18	63.82				1.2260
O K		0.525	2.62	7248.79	0.06	5.66				0.5405
Si K		1.739	1.37	6295.76	0.05	1.69				0.3258
Se K	(Ref.)	11.207	43.02	64383.26	0.38	18.83				1.0000
Pd K		21.121	30.82	7595.56	0.52	10.01				6.0721
Total			100.00			100.00				

Figure S8. EDS element mapping of fs laser-treated $PdSe_2$. (a) Cross-sectional element distribution, (b) EDS spectrum and (c) atomic percentage of C, Si, Pd, Se and O.

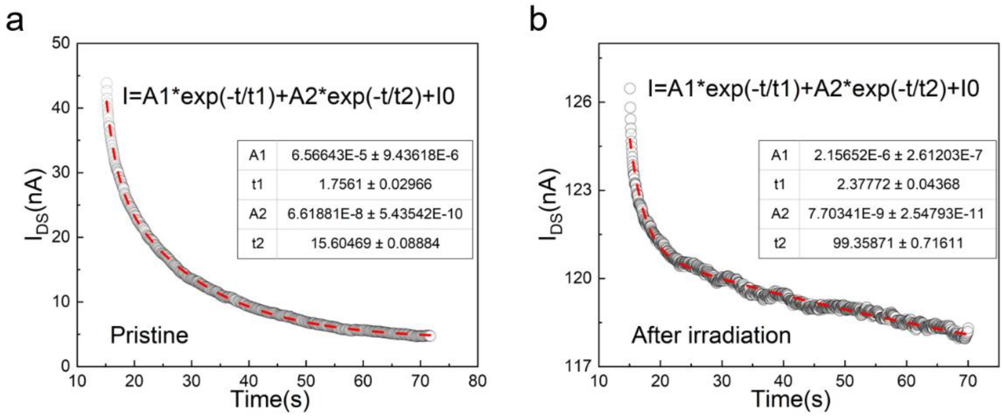

Figure S9. Double-exponential fitting of the current decay curves for (a) pristine and (b) defect-modified devices using formula: $I = I_0 + A_1 \times e^{-\frac{t}{\tau_1}} + A_2 \times e^{-\frac{t}{\tau_2}}$, fitting parameters of A_1, A_2, τ_1 and τ_2 are given in insets.

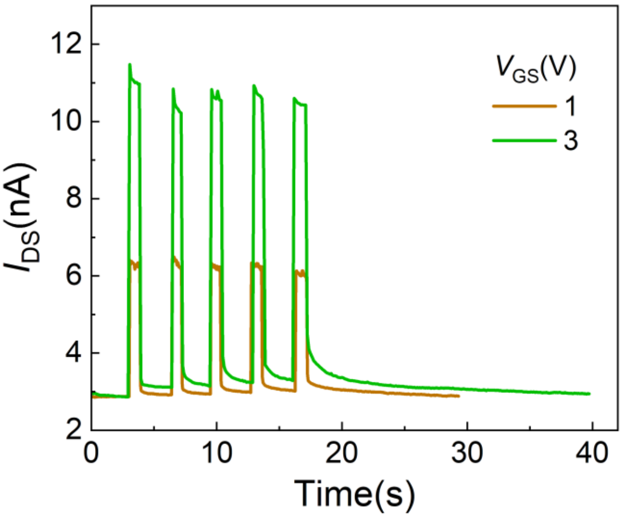

Figure S10. PSC of the device subjected to varied V_{GS} pulse amplitudes (1 and 3 V, 1 s).

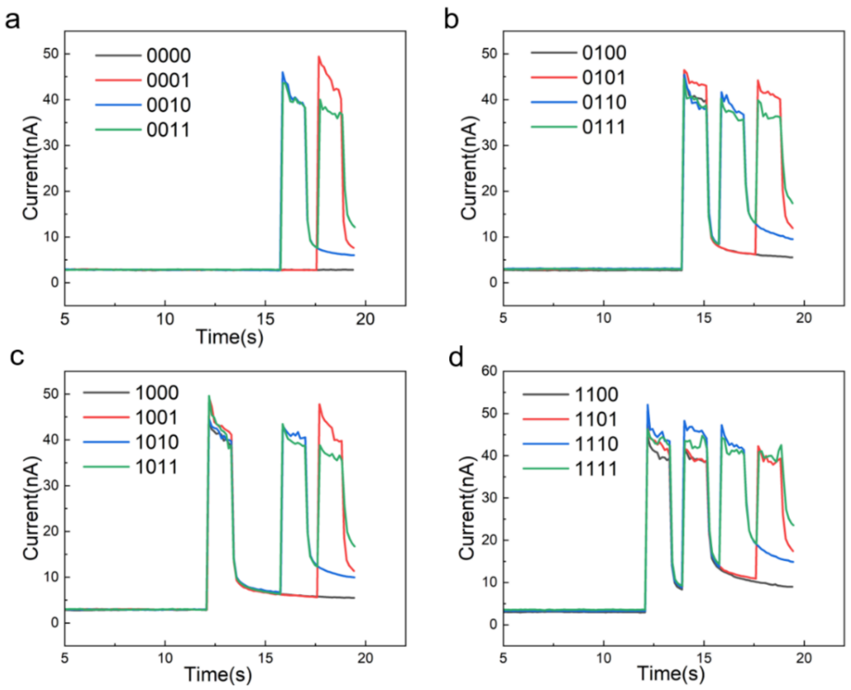

Figure S11. Output currents of the pristine $PdSe_2$ CTM for four-bit input pulse trains.

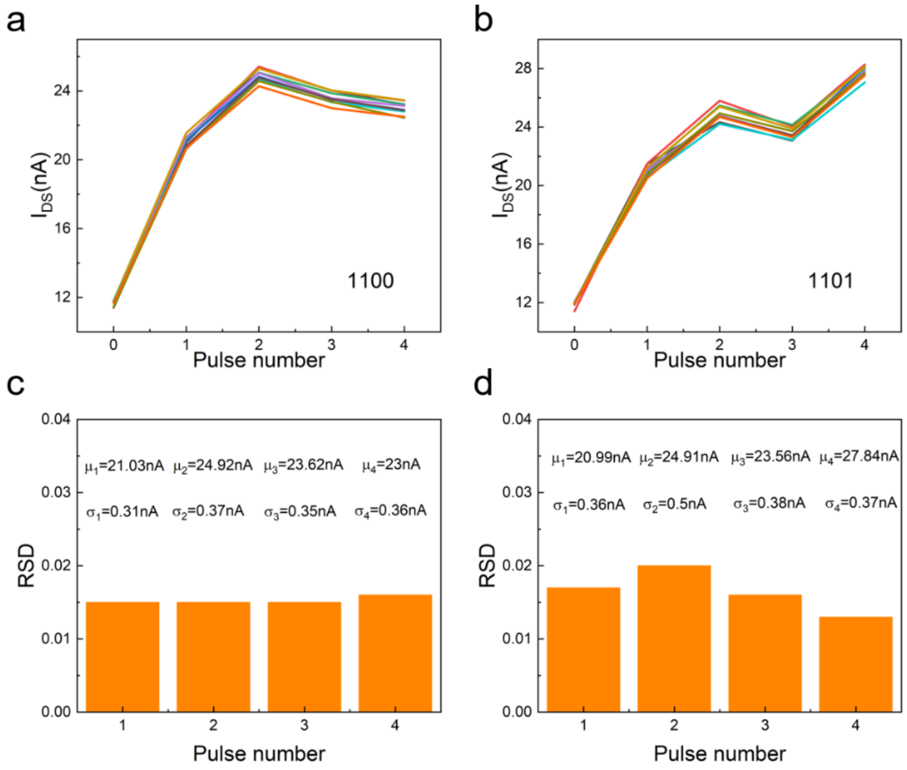

Figure S12. Current outputs to pulse sequences of (a) "1100" and (b) "1011", each for 10 cycles. (c) and (d) The average value (μ), standard deviation (σ), and relative standard deviation (RSD) of output currents for each consisting pulses are calculated.

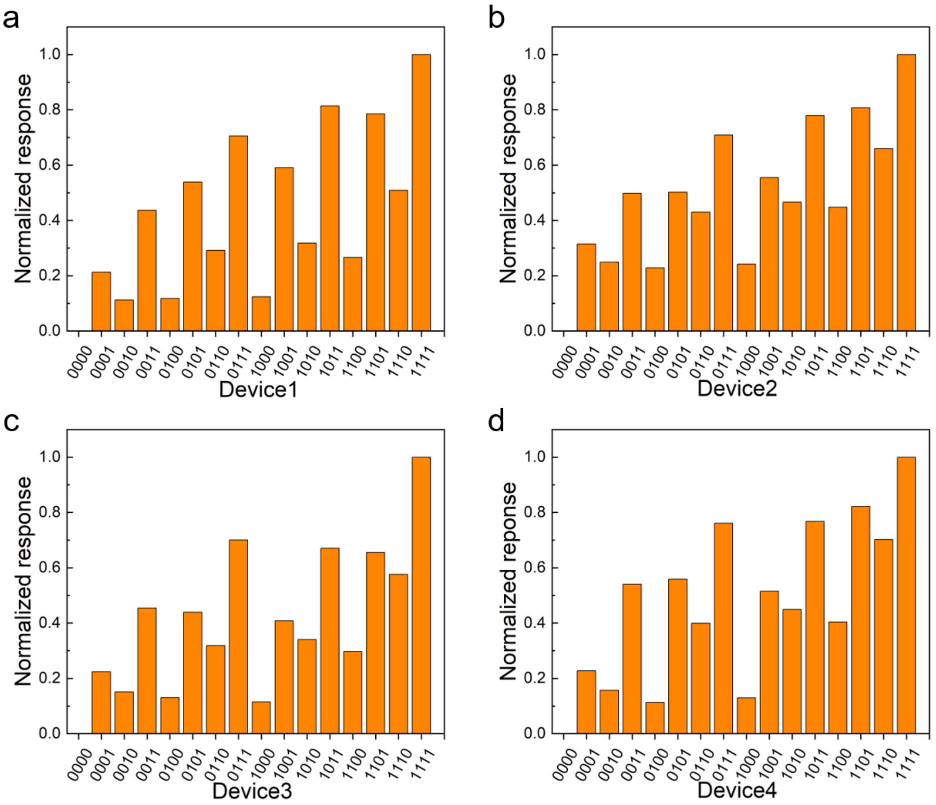

Figure S13. Normalized current responses of other four fabricated device with 16 physical reservoir states under four-bit inputs.

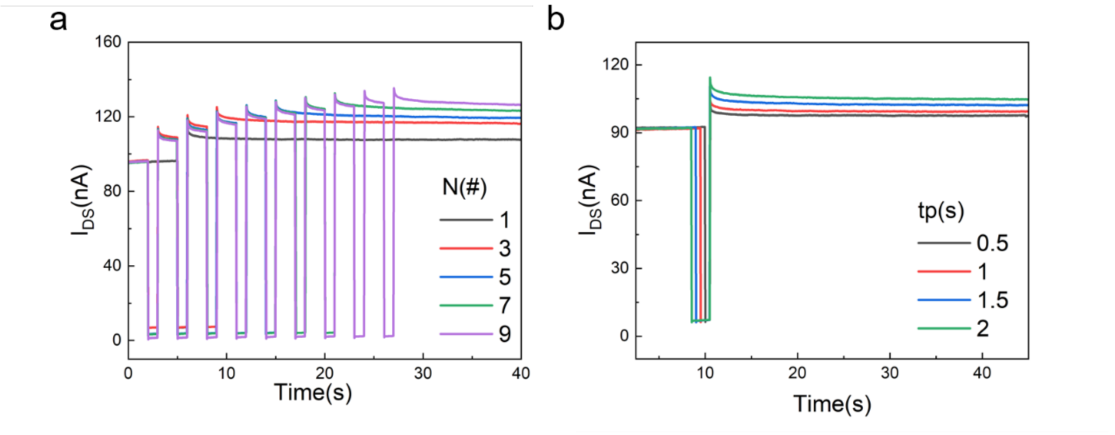

Figure S14. Response currents as a function of (a) pulse number (N) and (b) pulse duration (t_p) measured in defect-engineered $PdSe_2$ CTM.

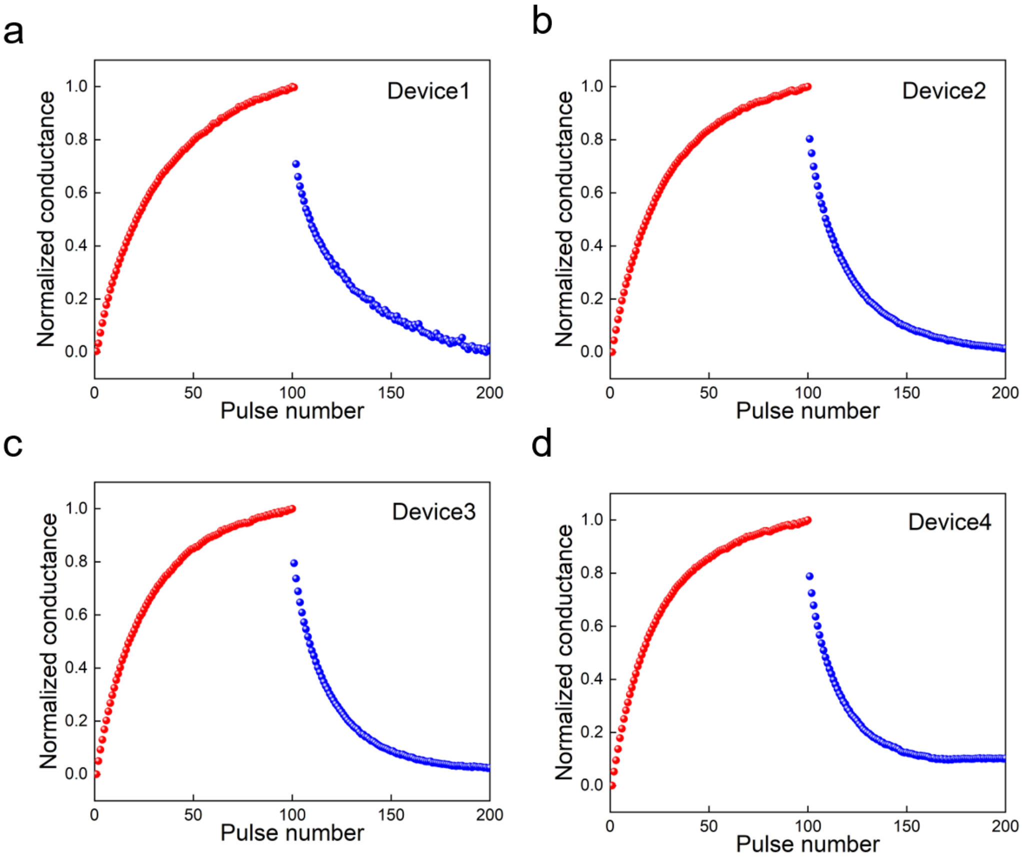

Figure S15. LTP/LTD characteristics of other four $PdSe_2$ CTMs with fs laser-induced defect engineering.

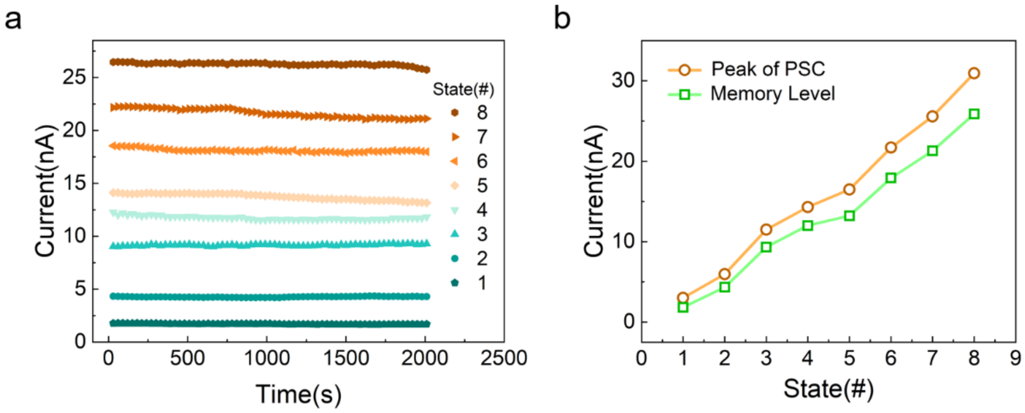

Figure S16. (a) Eight distinct current states and (b) their memory levels.

Table S1 Statistical characterization of the 8 current states

State / Statistics	1	2	3	4	5	6	7	8
μ(nA)	1.72	4.26	9.16	11.73	13.74	18.11	21.64	26.27
σ(nA)	0.035	0.040	0.074	0.173	0.303	0.164	0.432	0.139
RSD(#)	0.02	0.009	0.008	0.015	0.022	0.009	0.020	0.005

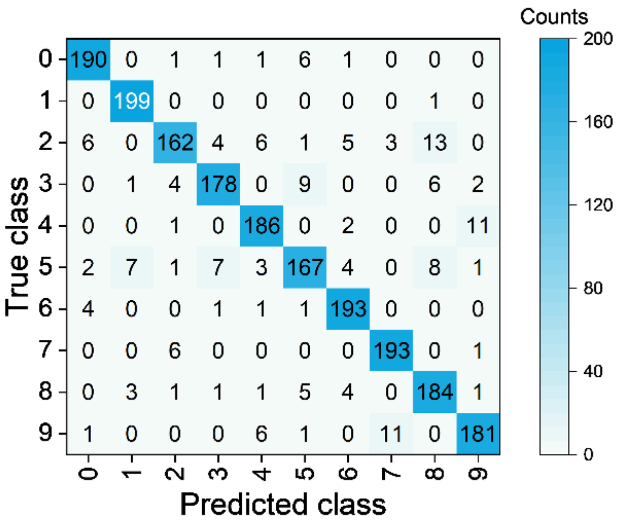

Figure S17. Confusion matrix for inference on 2000 test samples.

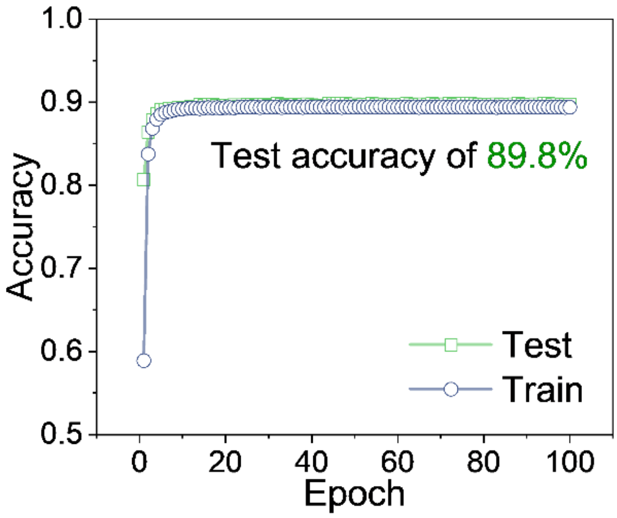

Figure S18. Accuracy for training and testing (inference) on full MNIST dataset.

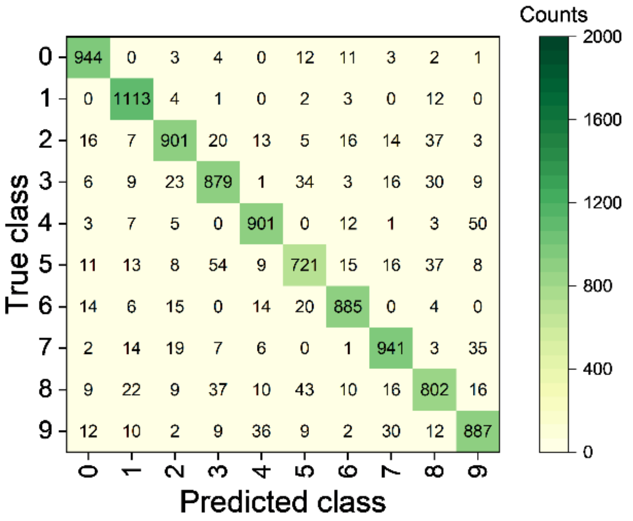

Figure S19. Confusion matrix for inference on 10000 test samples.

Supplementary Note S1: The mixed hardware-software implementation

Training and testing (inference) of the network was performed in Python software within a transfer interval that contains the measured current or conductance from device. The 4-bit binary pulses in the input RC layer are in one-to-one correspondence with the pulse sequence applied to the device, and there are 16 different combinations in total, that is, the values of the 16 reservoir states are normalized and mapped from the 16 corresponding device response currents. Meanwhile, each synapse of the readout layer contains the measured conductance values from two devices. The weight values of the synapses as well as the updated values are only obtained by looking up the table among the 128 values. Two devices with 64 measured conductances were mapped separately as the positive and negative ranges of the weight values. The mapping can be expressed as

$$\frac{\omega^{+}-\omega^{+}_{min}}{\omega^{+}_{max}-\omega^{+}_{min}} = G^{+}\frac{g^{+}-g^{+}_{min}}{g^{+}_{max}-g^{+}_{min}} \text{ and } \frac{\omega^{-}-\omega^{-}_{min}}{\omega^{-}_{max}-\omega^{-}_{min}} = G^{-}\frac{g^{-}-g^{-}_{min}}{g^{-}_{max}-g^{-}_{min}},$$

where *w* and *g* are the weight and conductance, respectively; the superscripts + and - denote the positive and negative values, respectively; the subscripts *max* and *min* represent the maximum and minimum values, respectively. *G* is the scaling factor usually set to 1.
